# Reference-guided MRI super-resolution with dual attention aggregation network

**DOI:** 10.3389/fneur.2026.1806780

**Published:** 2026-03-10

**Authors:** Lijuan Wang, Tao Chang, Lixiang Tan, Bin Shi, Yan Zhu

**Affiliations:** 1 Longdong University, Qingyang, China; 2 Lanzhou University, Lanzhou, China; 3 College of Artificial Intelligence and Computer Science, Northwest Normal University, Lanzhou, China; 4 Gansu Provincial Key Laboratory of Wearable Computing, School of Information Science and Engineering, Lanzhou University, Lanzhou, China; 5 Gansu Provincial Hospital, Lanzhou, China

**Keywords:** attention mechanism, deep learning, magnetic resonance imaging, reference-based super-resolution, vision transformer

## Abstract

Magnetic resonance imaging (MRI) super-resolution aims to enhance spatial resolution from low-resolution acquisitions while preserving anatomically meaningful structures. Conventional single-image super-resolution methods are fundamentally limited by the lack of high-frequency information in the input and often fail to recover fine anatomical details under large upscaling factors. In many MRI scenarios, additional reference images acquired under similar imaging protocols are naturally available and can provide complementary structural information, yet effectively leveraging such references without introducing anatomically inconsistent artifacts remains challenging. In this work, we propose a reference-guided MRI super-resolution framework, termed Dual Attention Aggregation Super-Resolution (DAASR), which explicitly balances structural modeling and controlled reference integration in a hierarchical manner. DAASR employs a channel-wise attention mechanism to reinforce global anatomical coherence in low-resolution features and a structure-aware alignment strategy to selectively incorporate consistent reference information while suppressing unreliable transfers. Extensive experiments on a public MRI benchmark and a clinical brain MRI dataset demonstrate that the proposed method consistently outperforms state-of-the-art MRI super-resolution approaches across multiple scaling factors in terms of PSNR and SSIM. The proposed DAASR further achieves improved structural similarity and visual consistency, indicating better preservation of fine anatomical structures and tissue boundaries.

## 1 Introduction

Magnetic resonance imaging (MRI) plays a crucial role in clinical diagnosis and biomedical research due to its excellent soft-tissue contrast and non-invasive nature (1, 2). However, the spatial resolution of MRI is inherently constrained by physical and practical factors, such as acquisition time, signal-to-noise ratio (SNR), and hardware limitations (3). High-resolution MRI acquisition often requires prolonged scanning, which may increase patient discomfort and susceptibility to motion artifacts. As a result, low-resolution MRI images are frequently acquired in clinical practice, leading to the loss of fine anatomical details that are important for accurate interpretation and quantitative analysis.

MRI super-resolution (SR) has emerged as an effective post-processing technique to enhance spatial resolution by reconstructing high-resolution images from low-resolution inputs. Early learning-based MRI SR methods primarily relied on single-image super-resolution (SISR) frameworks. SISR aims to recover a high-resolution (HR) output from a single low-resolution (LR) input. Although convolutional neural networks (CNNs) and, more recently, vision transformers have significantly advanced SISR, they fundamentally rely on a single degraded input. This reliance often results in blurred edges and loss of fine anatomical details when scaling factors are large, since crucial high-frequency details are absent in the LR observation (4–6).

In many MRI scenarios, however, additional reference images are naturally available. These references may originate from different contrasts of the same subject, adjacent slices, or scans acquired from other subjects under similar imaging protocols and anatomical conditions. Such reference images share consistent anatomical characteristics with the target image and can provide complementary structural information that is missing in the low-resolution input. Leveraging reference information for MRI super-resolution therefore represents a promising direction for improving reconstruction quality beyond the limits of single-image methods. Nevertheless, effectively incorporating reference images remains challenging. Inappropriate reference usage may introduce anatomically inconsistent details or amplify artifacts, which is particularly problematic in medical imaging applications where structural fidelity and interpretability are critical (7–9).

Existing reference-based or multi-contrast MRI SR methods attempt to exploit reference information through feature fusion, alignment, or contextual modeling. Although these approaches have shown encouraging results (10), they often face a trade-off between detail enhancement and reconstruction stability. Inaccurate alignment, insufficient structural modeling, or uncontrolled reference transfer may lead to degraded anatomical consistency, especially under high magnification factors or when reference images are not perfectly matched to the target anatomy. These limitations highlight the need for a reference-guided MRI SR framework that can selectively utilize complementary information while explicitly preserving global structural coherence (11–13).

To address the above challenges, we propose a Dual Attention Aggregation Super-Resolution (DAASR) framework for reference-guided MRI super-resolution. Unlike conventional approaches that directly transfer reference features, DAASR is designed to explicitly balance structural consistency and reference-guided detail enhancement in a hierarchical manner. The framework adopts a role-specialized dual-pathway design, in which the low-resolution (LR) branch focuses on modeling anatomically meaningful structures, while the reference branch provides complementary information under controlled alignment. These two pathways are coordinated through hierarchical attention mechanisms that progressively refine reconstruction quality while suppressing anatomically inconsistent reference cues. Specifically, a Channel-Wise Transformer (CWT) is introduced to process LR features in the channel domain, capturing long-range inter-channel dependencies that reinforce global anatomical coherence. To mitigate resolution and distribution discrepancies between the reference and target images, a Spatial Feature Extractor (SFE) encodes spatial information from both the reference image and its blurred counterpart, facilitating more reliable correspondence estimation. Building on these representations, the Spatial Alignment Transformer (SAT) performs structure-aware reference alignment using cross-attention, where LR structural features guide the selective integration of reference information. Patch-level index and confidence maps are further employed to emphasize anatomically consistent correspondences and suppress unreliable or mismatched transfers. These modules are integrated into stacked Hierarchical Feature Alignment Groups (HFAGs), enabling stable and progressive refinement of MRI reconstructions across multiple stages.

The main contributions of this work are summarized as follows:

1. We propose a reference-guided MRI super-resolution framework, termed DAASR, which explicitly balances anatomical structure preservation and controlled reference integration, effectively addressing the limitations of single-image MRI super-resolution under large upscaling factors.
2. We design a dual-attention aggregation strategy for reference-guided MRI reconstruction, in which a Channel-Wise Transformer models global anatomical structure while a structure-aware alignment mechanism selectively incorporates consistent reference information, reducing the risk of structurally inconsistent artifacts.
3. Extensive experiments on a public MRI benchmark and a clinical brain MRI dataset demonstrate that the proposed method consistently outperforms state-of-the-art MRI super-resolution approaches in terms of quantitative accuracy and anatomical interpretability, with comprehensive ablation studies validating the effectiveness of each key component.

## 2 Related works

### 2.1 Multi-contrast MRI super-resolution

Early studies on medical image super-resolution primarily focused on CNN-based models tailored for modality-specific or anisotropic MRI data. Oktay (14) introduced one of the earliest convolutional frameworks for cardiac MRI super-resolution using multi-input strategies. Subsequently, Du (15) addressed anisotropic 3D MRI reconstruction with residual CNNs, while Lyu (5) combined ensemble learning with complementary priors to improve generalization performance. Although these approaches demonstrated the feasibility of learning-based MRI super-resolution, they relied mainly on single-image cues and showed limited capability in exploiting cross-contrast structural information.

To overcome these limitations, multi-contrast MRI super-resolution (MCSR) methods were proposed to explicitly leverage reference contrasts acquired from the same subject or imaging protocol. Lyu (5) introduced a progressive framework to gradually integrate reference features into the target reconstruction. Feng (16) further proposed a multi-stage architecture that iteratively transfers high-frequency information across contrasts. More recently, Li (8) incorporated Transformer-based contextual matching and aggregation to model long-range dependencies between target and reference contrasts. Variational formulations were also explored by Lei (7) to improve reconstruction stability. Diffusion-based approaches, such as Disk-diff proposed by Mao (17), demonstrated improved perceptual quality at the cost of significantly increased computational complexity. In parallel, clinical-oriented studies have emerged, including the work of Li (18), which employed GANs with attention mechanisms and cyclic losses for pelvic MRI reconstruction.

Despite steady progress, existing MCSR methods still face challenges in balancing reconstruction fidelity and stability. CNN-based models often produce over-smoothed results that obscure fine anatomical structures such as sulci and cortical ribbons, while Transformer- or diffusion-based methods may amplify artifacts or impose heavy computational burdens.

In contrast to these approaches, our method aims to leverage reference information in a more controlled and structure-aware manner, focusing on preserving anatomically meaningful structures while maintaining stable reconstruction behavior across different MRI datasets and resolution scales.

### 2.2 Reference-based super-resolution

Reference-based super-resolution (RefSR) enhances image reconstruction by incorporating an additional high-resolution reference image that provides complementary information beyond the low-resolution input. In general computer vision tasks, RefSR methods exploit texture-rich references that are aligned in content or semantics with the target image, obtained through external image retrieval or neighboring frames in multi-view or video settings (19–22).

Existing RefSR approaches can be broadly categorized into pixel-alignment–based and patch-matching–based methods. Pixel-alignment–based methods attempt to directly register the reference image to the target using global transformation or dense correspondence estimation. For example, Landmark (23) employed energy minimization for global alignment, Zheng (24) estimated optical flow for cross-scale feature fusion, and Ronggui (25) utilized deformable convolution to enable adaptive warping. While effective under limited disparity, these approaches are sensitive to misalignment and often degrade when large structural differences exist between the reference and target.

Patch-matching–based methods alleviate strict alignment requirements by transferring reference information at the feature level. Zhang (26) introduced multi-scale patch matching in a deep feature space, followed by feature swapping to enrich textures. Lu (27) proposed a coarse-to-fine matching strategy with spatial adaptation to mitigate distribution gaps. Yang (28) adopted a Transformer architecture with hard and soft attention mechanisms to select relevant reference features. Jiang (29) further improved correspondence quality using contrastive learning and teacher–student distillation, while Xie (30) avoided irrelevant semantic priors by learning SR-specific features. More recently, multi-reference RefSR methods such as Yan (31) and Pesavento (32) explored matching and fusing information from multiple reference images to increase texture diversity.

Although RefSR methods have achieved impressive results on natural images, they often encounter two critical issues: reference underuse, where insufficient information is extracted from the reference, and reference misuse, where mismatched or inconsistent features are transferred. These issues are particularly problematic in medical imaging, where anatomically incorrect details may compromise structural interpretability.

Motivated by these challenges, our work adapts reference-based super-resolution to the MRI domain by emphasizing structure-aware alignment and controlled reference integration, aiming to exploit complementary information while suppressing anatomically inconsistent cues.

## 3 Methods

This section offers a comprehensive explanation of the proposed Dual Attention Aggregation Super-Resolution (DAASR) network. We first present an overview of the overall structure of DAASR. The network’s core is composed of two transformer modules connected in series, the Channel-Wise Transformer (CWT), introduced in Section 3.2, which processes the low-resolution (LR) input; and the Spatial Alignment Transformer (SAT), described in Section 3.4, which refines features using the reference (Ref) image. Section 3.3 focuses on the feature extraction process for the reference image, while Section 3.5 presents the loss functions used to optimize the network.

### 3.1 Overview

**Figure 1:**
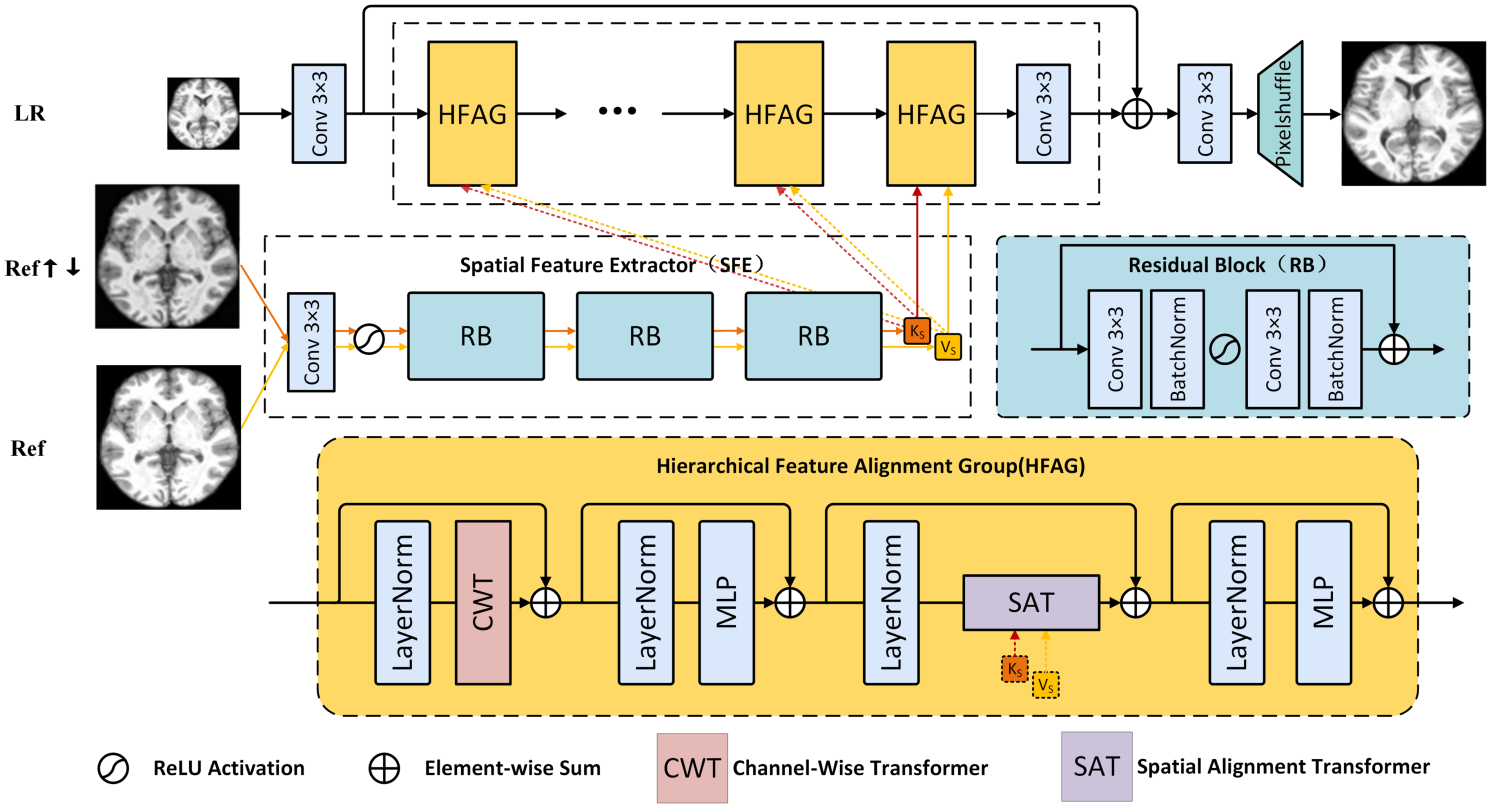
The proposed DAASR structure for RefSR. It consists of a structure model branch (via CWT) and a texture transfer branch (via SFE and SAT), enabling accurate feature alignment and high-frequency detail recovery.

As illustrated in Figure 1, the proposed DAASR framework is designed for reference-guided MRI super-resolution at the slice level. Given a low-resolution (LR) MRI slice and a reference (Ref) MRI slice acquired from the same imaging domain, the network aims to reconstruct a high-resolution (HR) image while preserving anatomically meaningful structures.

The LR MRI slice is first processed by a shallow feature extraction module to obtain initial representations that encode basic anatomical layouts. In parallel, the reference MRI slice is processed by a dedicated feature extractor to generate reference features that capture complementary structural information. Instead of directly transferring reference features, DAASR introduces a controlled integration strategy to ensure that only anatomically consistent information contributes to the reconstruction.

The core of the framework is the Hierarchical Feature Alignment Group (HFAG), which is stacked multiple times to progressively refine MRI feature representations. Each HFAG consists of two transformer-based modules connected in series: the Channel-Wise Transformer (CWT) and the Spatial Alignment Transformer (SAT). CWT focuses on modeling global inter-channel dependencies within MRI features, which helps reinforce coherent anatomical structures across the slice. SAT subsequently aligns reference features with the LR features in a structure-aware manner, guiding the recovery of fine anatomical details while suppressing mismatched reference cues.

By alternately performing structural modeling and reference-guided alignment within each HFAG, the proposed framework incrementally improves reconstruction quality and stabilizes anatomical consistency. After multiple refinement stages, the aggregated features are upsampled to the HR space to produce the final reconstructed MRI image.

As shown in Equation 1 within each HFAG, the input feature $F_{\text{in}}$ from the LR branch is first processed by CWT to extract structure-preserving features. These are then aligned and enriched with reference textures through SAT, forming an output feature map that simultaneously respects LR geometry and incorporates reliable high-frequency details.


$$F_{\mathrm{CWT}}=\mathrm{CWT}(F_{\text{in}})$$ (1)

The CWT specializes in modeling inter-channel dependencies and enhancing semantic consistency, yielding the structural feature map $F_{\mathrm{CWT}}$ .

Next, the reference image $I_{\mathrm{Ref}}$ and its blurred counterpart $I_{\mathrm{Ref}}^{↑↓}$ (obtained by 4 × bicubic upsampling and downsampling) are processed through a shared spatial feature extractor to obtain $F_{\mathrm{Ref}}$ and $F_{\mathrm{Ref}}^{↑↓}$ . These, along with $F_{\mathrm{CWT}}$ , are fed into the SAT module.


$$F_{\mathrm{SAT}}=\mathrm{SAT}(F_{\mathrm{CWT}},F_{\mathrm{Ref}},F_{\mathrm{Ref}}^{↑↓})$$ (2)

Inside SAT, we first compute the resemblance between the query $Q_{S}$ from $F_{\mathrm{CWT}}$ and the key $K_{S}$ from the reference branch $F_{\mathrm{Ref}}^{↑↓}$ , resulting in an index map P and a similarity score map C. Using the index map, the most significant texture features are retrieved from the reference, then fused with the structure features $F_{\mathrm{CWT}}$ . The fused result is multiplied by C to obtain the final texture-enhanced representation $F_{\mathrm{SAT}}$ (Equation 2).

Through Equation 3, the final output of the group is formed via residual aggregation.


$$F_{\text{Out}}=F_{\mathrm{CWT}}+F_{\mathrm{SAT}}+F_{\text{in}}$$ (3)

This formulation ensures efficient feature propagation and allows multiple HFAGs to be stacked for progressive refinement.

### 3.2 Channel-wise transformer

**Figure 2:**
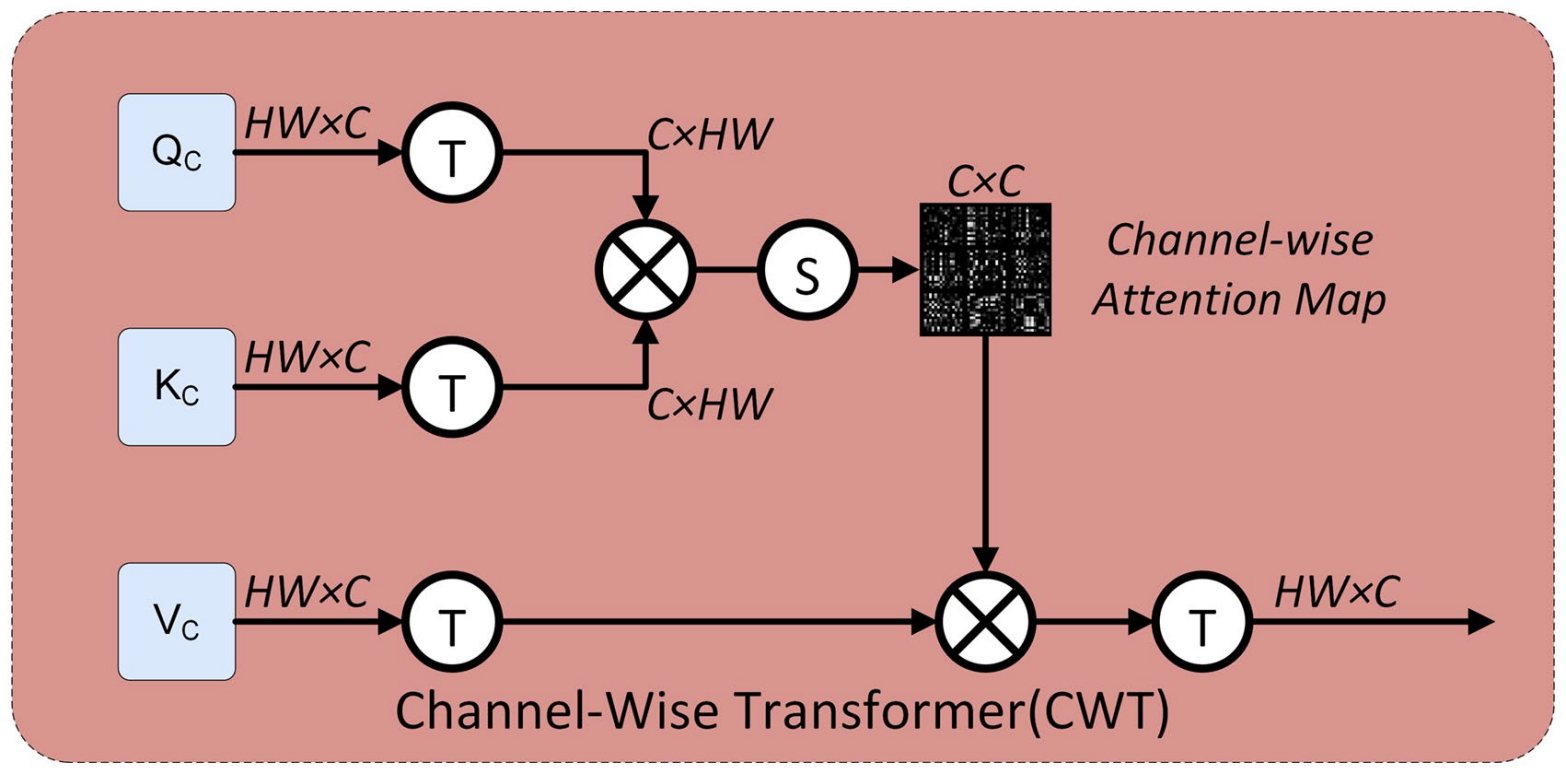
Internal structure of the Channel-Wise Transformer (CWT). The Q, K, and V features are reshaped along the channel axis, forming a C×C attention map that captures inter-channel dependencies. This attention is then applied to value features, followed by transposition and residual fusion.

To perform structure-aware SISR on the LR input, we adopt a transformer block composed of the sequence, LN-CWT- LN-MLP, following designs proven effective in previous transformer-based SR methods. As shown in Figure 2, the input feature map of size H×W×C is first reshaped to enable attention computation in the channel domain. Specifically, the spatial dimensions H×W are flattened, and the feature tensor is rearranged into a two-dimensional matrix of shape (HW)×C . This operation ensures that each column corresponds to a feature channel, while each row encodes the spatial responses within that channel. By applying linear projections, we obtain the query ( $Q_{C}$ ), key ( $K_{C}$ ), and value ( $V_{C}$ ) matrices, all represented as HW×C . To transform attention from the spatial domain to the channel domain, the matrices are further transposed, resulting in C×HW . This reshaping allows the subsequent dot-product operation to generate a C×C attention map, which explicitly models global dependencies between channels, rather than local correlations across pixels.

In the Channel-Wise Transformer module, we focus on modeling inter-channel dependencies. While Equation 4 let the input feature be $F_{\text{in}}\in R^{H\times W\times C}$ , which is reshaped into a 2D matrix $\hat{F_{\text{in}}}\in R^{\mathrm{HW}\times C}$ . Next, the matrices for query, key, and value are obtained via linear projections and then transposed.


QC=TFin⋅WQ,KC=TFin⋅WK,VC=TFin⋅WV (4)

Where $W_{Q},W_{K},W_{V}\in R^{C\times C}$ are learnable parameters, and T(⋅) denotes the transpose operation. This step first maps the spatially arranged features into the attention embedding space, and then transposes the matrices to move attention computation from the spatial to the channel domain, resulting with $Q_{C},K_{C},V_{C}\in R^{C\times \mathrm{HW}}$ for channel-wise attention.

The channel attention is computed as Equation 5


$$F_{\mathrm{CWT}}=T^{-1}(A(Q^{C},K^{C},V^{C}))=\text{Softmax}(Q_{C}{K_{C}}^{T})\cdot V_{C}$$ (5)

Where $T^{-1}(\cdot )$ denotes the inverse transpose operation that restores the output shape to match the spatial alignment of the input $R^{\mathrm{HW}\times C}$ , for further processing.

Unlike SwinIR, which performs self-attention over spatial tokens, or SENet, which aggregates channel responses via global pooling, our design applies attention directly in the channel dimension by transposing Q, K, and V matrices. This explicit channel-wise modeling allows the network to better capture global structural dependencies. By explicitly modeling inter channel relationships, the CWT module improves the LR feature’s semantic encoding. This design enables the model to capture long-range inter-channel dependencies that are overlooked in conventional attention schemes. As a result, CWT provides a more principled solution for preserving structural fidelity in LR-guided features, offering a clear advantage over both CNN-based encoders and existing spatial or channel attention modules.

### 3.3 Spatial feature extractor

In the DAASR framework, the reference feature extraction module serves as a pivotal transition between structural modeling (via CWT) and texture transfer (via SAT). Its primary role is to encode spatial texture features from both the reference image and its blurred variant, which are then utilized as the Key and Value inputs to the subsequent SAT module for cross-image correspondence modeling and high-frequency detail transfer.

In reference-based super-resolution (RefSR), accurate and adaptive extraction of spatial textures from the reference image is critical for restoring high-fidelity outputs. Conventional approaches often employ pre-trained VGG networks to obtain shallow texture features. However, VGG was originally designed for semantic abstraction in image classification and does not align well with the low-level texture modeling required in RefSR. Furthermore, fixed pre-trained weights lack adaptability to diverse image domains or resolution gaps. To overcome these limitations, we design a lightweight and task-specific Spatial Feature Extractor (SFE). SFE consists of a convolutional layer, an activation function, and three Residual Blocks (RB). Each residual block (RB), illustrated in Figure 1, consists of two 3×3 convolutional layers followed by batch normalization and ReLU activation, with a skip connection to stabilize training. This lightweight design enhances feature expressiveness while maintaining efficiency, enabling the SFE to better capture fine-grained texture representations from both the reference and its blurred counterpart.

Given a reference image $I_{\mathrm{Ref}}$ and its blurred counterpart $I_{\mathrm{Ref}}^{↑↓}$ we apply the SFE module to both and obtain texture embeddings $F_{\mathrm{Ref}}$ and $F_{\mathrm{Ref}}^{↑↓}$ . These are used to construct the Key and Value in the SAT module. Meanwhile, the output of the CWT $F_{\mathrm{CWT}}$ is upsampled and projected into the Query representation. This interaction is formulated as.


$$Q_{S}=\mu (F_{\mathrm{CWT}})\cdot \overset{´}{W_{Q}},K_{S}=\mathrm{SFE}(F_{\mathrm{Ref}}^{↑↓}),V_{S}=\mathrm{SFE}(F_{\mathrm{Ref}})$$ (6)

Where μ(⋅) denotes an upsampling function and $\overset{´}{W_{Q}}\in R^{C\times C}$ is a learnable projection matrix. Unlike prior methods such as TTSR (33) that jointly process LR and Ref images through a shared extractor to reduce distribution mismatch, we decouple structure and texture modeling into separate branches, LR features are processed via CWT to focus on inter-channel structure, while reference features are extracted independently via SFE to model spatial textures. This design facilitates precise attention-based matching and enables clearer texture transfer.

To address the resolution gap, the structural features from CWT are upsampling before entering SAT. To tackle the distribution gap, the original and blurred reference images are processed separately via the SFE module. This dual-path strategy allows the SAT to perform more accurate reference indexing and feature selection. Finally, the extracted texture representations are fused with structural features and modulated by a learned similarity confidence map, completing the texture enhancement phase.

### 3.4 Spatial alignment transformer layer

**Figure 3:**
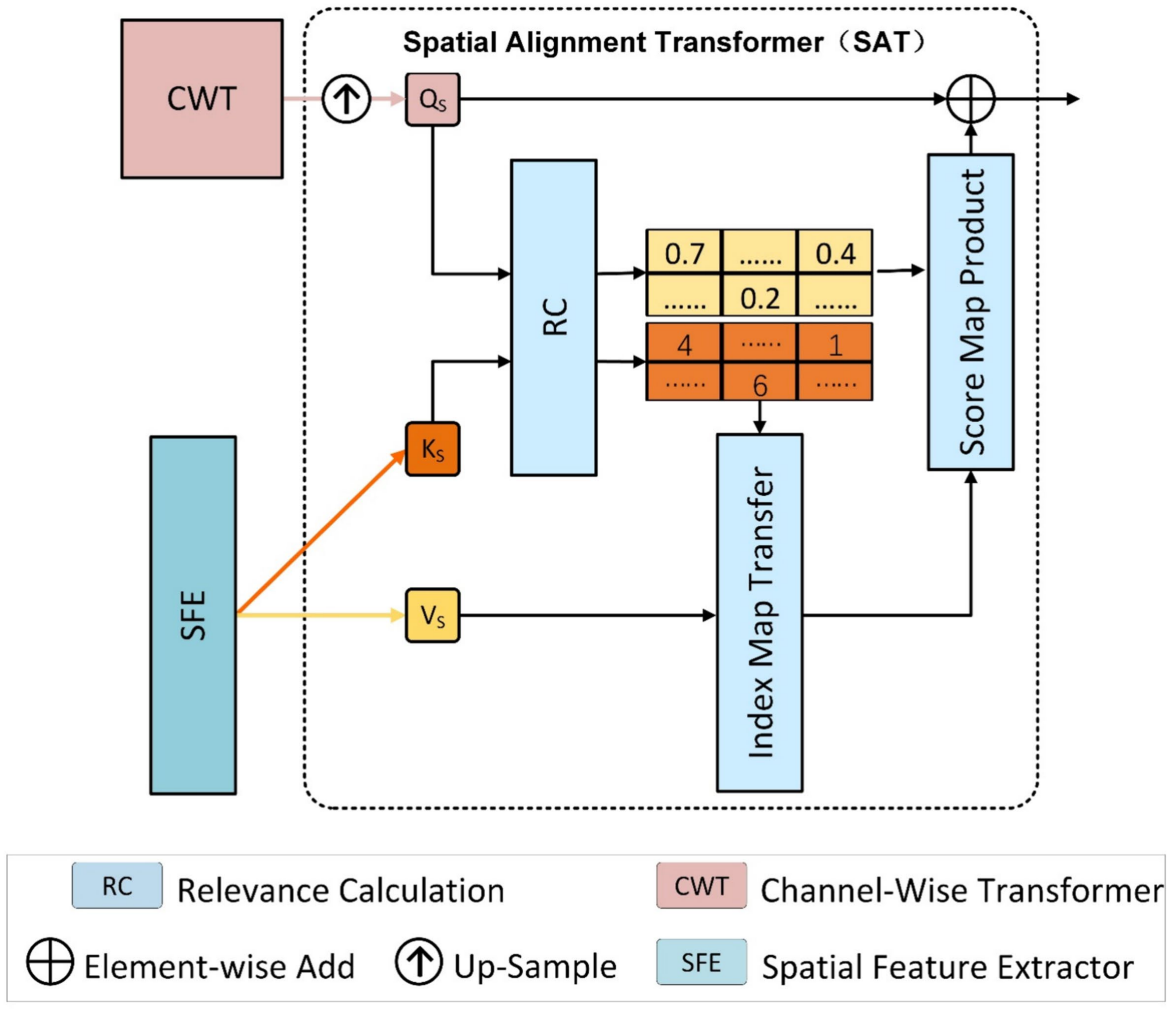
Internal structure of the spatial alignment transformer (SAT). SAT module addresses this by selecting and aggregating spatial features most relevant to the LR content, based on patch-level similarity between query and key features.

In reference-based super-resolution, transferring high-frequency texture details extracted from the reference image is essential for recovering fine details lost in the low-resolution input. As shown in Figure 3. The Spatial Alignment Transformer (SAT) module addresses this by selecting and aggregating spatial features most relevant to the LR content, based on patch-level similarity between query and key features.

Given the extracted embeddings $Q_{S}\in R^{N\times C}$ , $K_{S}\in R^{M\times C}$ , $V_{S}\in R^{M\times C}$ based on Equation 6, we first compute a similarity matrix $M_{i,j}\in R^{N\times M}$ using normalized dot-product (i.e., cosine similarity) with Equation 7


$$M_{i,j}=\langle \frac{q_{i}}{\left\|q_{i}\right\|},\frac{k_{j}}{\left\|k_{j}\right\|}\rangle ,\text{for}\;i\in [1,N],j\in [1,M]$$ (7)

Where $q_{i}\;$ and $k_{i}$ are flattened patches from $Q_{S}$ and $K_{S}$ , respectively.

For each query patch $q_{i}$ , we derive a relevance calculation index map P and a confidence score map C by selecting the most relevant matching response as shown in Equation 8, 9


$$p_{i}=\arg\max_{j}M_{i,j}$$ (8)

$$c_{i}=\max_{j}M_{i,j}$$ (9)

Where, $p_{i}$ indicates the index of the most relevant patch in $K_{S}$ , and $c_{i}$ is the corresponding similarity score. Instead of aggregating over all values via product (which often introduces blur), we directly sample the most relevant patch from $V_{S}$ using P, and concatenate it with the LR structure feature $F_{\mathrm{CWT}}$ to obtain


$$\overset{´}{V}=[F_{\mathrm{CWT}},{\left(V_{S}\right)}_{P}]$$ (10)

Concatenated feature $\overset{´}{V}\in R^{N\times 2C}$ based on Equation (10), the output undergoes refinement via a 1 × 1 convolutional operation, and is then combined with the confidence score map through element-wise product


$$F_{\mathrm{SAT}}=\text{Conv}\;(\overset{´}{V})⊙C$$ (11)

Where ⨀ is the element-wise product.

This mechanism allows SAT to selectively transfer sharp texture details with high semantic relevance, while avoiding the over smoothing effects commonly observed in element-wise product. Our P–C design unifies the strengths of both hard retrieval and soft weighting, ensuring that only reliable reference patches are transferred while suppressing spurious textures. This dual mechanism offers a novel and more robust approach to reference alignment than existing RefSR pipelines. As a result, the output $F_{\mathrm{SAT}}$ based on Equation 11 provides high-fidelity texture representations that complement the structural features from CWT and contribute to the overall enhancement in HFAG.

### 3.5 Loss function

To ensure stable and effective training of the DAASR, we implement a hierarchical loss design that supervises the model at three levels, structure modeling (CWT), texture transfer (SAT), and final SR reconstruction. Each module receives a task-specific objective, and all are unified through a total weighted loss.

(1) Structure Loss for CWT. The CWT module is responsible for modeling structural features from the LR image in the channel domain. To ensure its independent reconstruction capability, we use a pixel-level L1 loss to its output $I_{\mathrm{CWT}}$ on Equation 12

$$L_{\mathrm{CWT}}=\|I_{\mathrm{CWT}}-I_{\mathrm{HR}}\|$$ (12)

(2) Texture Loss for SAT. The SAT module focuses on transferring spatial textures from the reference image. In addition to an L1 loss, we introduce a perceptual loss to capture richer detail representations. As shown in Equation 13 specifically, we extract VGG-19 features from the relu3_3 layer as follows

$$L_{\mathrm{SAT}}=\|I_{\mathrm{SAT}}-I_{\mathrm{HR}1}\|+\lambda_{p}\cdot {\left\|\phi (I_{\mathrm{CWT}})-\phi (I_{\mathrm{HR}})\right\|}_{2}^{2}$$ (13)

Where ϕ(⋅) denotes the relu3_3 activation features from the pretrained VGG-19 network, and $\lambda_{p}=10^{-1}$ controls the perceptual term. This additional supervision complements our lightweight SFE module and enhances high-frequency fidelity during training. It should be noted that the perceptual term is used only as an auxiliary regularization during training and does not dominate the reconstruction objective, thereby avoiding the introduction of anatomically implausible structures.

(3) Final Reconstruction Optimization. The final output $I_{\mathrm{SR}}$ , obtained by aggregating structure and texture information through HFAG, represents the primary prediction of our network. An L1 loss is used to supervise the difference between the SR output and the reference $I_{\mathrm{HR}}$ , ensuring consistency in both local and global reconstruction goals. The overall optimization criterion is expressed as a weighted aggregation of the components outlined above on Equation 14.

$$L=\lambda_{1}\cdot {\left\|I_{\mathrm{SR}}-I_{\mathrm{HR}}\right\|}_{1}+\lambda_{2}\cdot L_{\mathrm{CWT}}+\lambda_{3}\cdot L_{\mathrm{SAT}}$$ (14)

We empirically set the weights to $\lambda_{1}=1$ , $\lambda_{2}=10^{-1}$ , and $\lambda_{3}=10^{-2}$ to prioritize final reconstruction quality, while still guiding the intermediate modules toward structured and detailed feature representations.

## 4 Result

### 4.1 Datasets and evaluation metrics

#### 4.1.1 Datasets

To comprehensively evaluate the performance of the proposed reference-based super-resolution framework for MRI reconstruction, experiments are conducted on two datasets, including a public benchmark dataset (FastMRI) (33) and a clinical brain MRI dataset collected from Gansu Provincial People’s Hospital (denoted as Brain (ours)). These datasets cover different anatomical regions and acquisition conditions, enabling a robust assessment of the model’s generalization ability and anatomical fidelity.

FastMRI is a large-scale, publicly available MRI dataset released by Facebook AI Research, widely adopted for developing and benchmarking learning-based MRI reconstruction and super-resolution methods. For FastMRI, we randomly select 200 volumes for training and validation following the standard data preparation protocol. For the reference-based setting, a random HR image is selected from the dataset as the reference image for each LR input. The selected reference image is further down-sampled using the same degradation process to form an LR–Ref pair, ensuring consistency between the LR input and the reference while avoiding trivial identity mapping.

The Brain (ours) dataset is a clinical brain MRI dataset collected from Gansu Provincial People’s Hospital, approved by the institutional review board with all patient-identifying information removed prior to analysis. The dataset consists of multi-slice brain MRI scans acquired under routine clinical protocols. All images are anonymized and used solely for research purposes. The dataset yields a total of 150 axial brain MRI slices. Each slice is resized or cropped to 256 × 256 resolution for training and evaluation. To assess the robustness of super-resolution under different anatomical conditions, both skull-stripped and non–skull-stripped brain images are included in the experiments. Skull stripping is performed using a commonly used automated brain extraction pipeline, allowing us to evaluate the model’s ability to reconstruct fine anatomical structures both with and without surrounding cranial context. Low-resolution images are generated by applying bicubic down-sampling to the HR images with scaling factors of ×2, ×3, and ×4. For reference-based super-resolution, a random HR image is selected from the Brain (ours) dataset as the reference image for each LR input. The reference image is then down-sampled using the same degradation operation to form a corresponding LR–Ref pair. This strategy simulates practical scenarios in which reference images are drawn from the same acquisition domain but are not strictly aligned with the target image.

For each dataset, we construct non-overlapping train/validation/test splits and report results on the held-out test split only. Unless otherwise specified, all hyperparameters are selected on the validation split. We use the same split protocol for all compared methods to ensure a fair evaluation.

For the Brain (ours) dataset, the split is performed at the subject/volume level to keep slices from the same subject within the same split.

#### 4.1.2 Evaluation metrics

To quantitatively assess the reconstruction quality of the super-resolved MRI images, we primarily adopt two widely used objective evaluation metrics: Peak Signal-to-Noise Ratio (PSNR) and Structural Similarity Index Measure (SSIM). PSNR measures the pixel-wise fidelity between the reconstructed super-resolution image and the ground-truth high-resolution (HR) image, reflecting the overall reconstruction accuracy. A higher PSNR value indicates a smaller reconstruction error. SSIM evaluates the structural similarity between two images by jointly considering luminance, contrast, and structural information. Compared with PSNR, SSIM better correlates with structural consistency, which is particularly important for MRI images where anatomical integrity is critical for clinical interpretation.

Specifically, PSNR is defined as Equation 15


$$\text{PSNR}=10\log_{10}(\frac{\mathrm{MA}X^{2}}{\mathrm{MSE}}),$$

$$\mathrm{MSE}=\frac{1}{N}\sum_{1=1}^{N}{\left(I_{i}^{\mathrm{SR}}-I_{i}^{\mathrm{HR}}\right)}^{2}$$ (15)

Where *MAX* denotes the maximum possible intensity value (after normalization) and *N* is the number of pixels. SSIM is computed as Equation 16


$$\text{SSIM}(x,y)=\frac{\left(2u_{x}u_{y}+C_{1})(2\sigma_{\mathrm{xy}}+C_{2}\right)}{\left(u_{x}^{2}+u_{y}^{2}+C_{1})(\sigma_{x}^{2}+\sigma_{y}^{2}+C_{2}\right)}$$ (16)

Where $u_{x},u_{y}$ are mean intensities, $\sigma_{x}^{2},\sigma_{y}^{2}$ are variances, and $\sigma_{\mathrm{xy}}$ is the covariance between the SR image *x* and HR image *y*; $C_{1},C_{2}$ are small constants for numerical stability.”

In addition to PSNR and SSIM, we report the Learned Perceptual Image Patch Similarity (LPIPS) as an auxiliary evaluation metric in the ablation study of loss function design. LPIPS measures the perceptual similarity between images in a deep feature space, with lower values indicating higher similarity. It is used in this work solely to reflect subtle appearance differences induced by different loss weight settings and does not serve as a primary or clinical evaluation criterion. The main quantitative evaluation of MRI super-resolution performance throughout this paper remains focused on PSNR and SSIM, which are more directly related to anatomical fidelity and reconstruction accuracy. Following common practice, boundary pixels are cropped before evaluation to avoid border effects introduced by convolutional processing.

### 4.2 Implementation details

The proposed DAASR adopts a stacked architecture, where the core module—Hierarchical Feature Alignment Group (HFAG)—is repeated 6 times to progressively refine structure and texture representations through dual attention modeling. This hierarchical design enables the network to alternately enhance structural consistency and reference-guided detail reconstruction, which is particularly beneficial for preserving anatomical integrity in MRI super-resolution tasks. The model is trained using the PyTorch framework on a single NVIDIA RTX 4070ti Super GPU. We use a batch size of 9, and train for a total of 200 epochs. The initial learning rate is set to 2 × 10^−4^, and reduced by half every 20 epochs using a step decay schedule. The network is optimized with the Adam optimizer, where the default parameters are set to *β*₁ = 0.9, β₂ = 0.999. We adopt an adversarial learning strategy to encourage realistic high-frequency recovery while controlling potential artifact amplification in MRI reconstructions. Both the generator and discriminator are updated with a learning rate of 1 × 10^−4^. Based on validation and prior work, the loss weights are set to *λ*₁ = 1 (final reconstruction loss), λ₂ = 10^−1^ (structure loss for CWT), and λ₃ = 10^−2^ (texture loss for SAT).

When adversarial learning is enabled, we use a standard GAN objective for MRI SR with a convolutional discriminator operating on image patches. The discriminator is trained to distinguish the reconstructed SR images from the HR targets, while the generator is optimized jointly with the reconstruction and perceptual/texture losses. We keep the generator and discriminator learning rates at 1 × 10^−4^ and use the same adversarial configuration across all scales.

During training, all MRI images are processed in a slice-based manner, where each 2D slice is treated as an independent sample. All slices are resized to a spatial resolution of 256 × 256 for unified processing. Low-resolution (LR) images are generated by applying bicubic down-sampling to the corresponding high-resolution (HR) images with scaling factors of ×2, ×3, and ×4. This setting is widely adopted in MRI super-resolution studies and allows consistent evaluation across different anatomical regions and datasets. To enhance the robustness of the model and mitigate overfitting, standard data augmentation strategies are employed during training, including random horizontal and vertical flipping as well as rotations by 90°, 180°, and 270°. These augmentations are applied consistently to both the LR inputs and their corresponding reference images to maintain spatial correspondence.

For the reference-based super-resolution setting, each LR input is paired with a reference image randomly selected from the same dataset. The selected reference image is down-sampled using the same degradation process to form an LR–Ref pair. This strategy avoids trivial identity mapping while simulating realistic clinical scenarios, in which reference images share similar anatomical characteristics with the target image but are not spatially aligned. All competing methods are trained and evaluated under the same experimental protocol whenever possible, including identical data splits, input resolutions, and evaluation metrics. To avoid any ambiguity, reference images are sampled from the same split as the query LR image (train/val/test), and we do not retrieve references across splits.

We compute PSNR and SSIM on the luminance-equivalent intensity space following a consistent preprocessing pipeline across all methods. To ensure fair comparison, we apply the same border handling/cropping rule for all models at each scaling factor. For reference-guided settings, the reference image for each LR input is sampled from the corresponding split (train/val/test) only, and we do not allow reference retrieval across splits. This protocol is used for all quantitative results reported in Table 1.

**Table 1:** Quantitative results on two datasets with different enlargement scales, in terms of SSIM and PSNR.

Scale		2×		3×		4×	
Dataset	Method	PSNR	SSIM	PSNR	SSIM	PSNR	SSIM
FastMRI	Bicubic	25.26	0.6921	24.91	0.6871	24.24	0.6804
	SRCNN (34)	28.74	0.7324	28.48	0.7124	28.19	0.7101
	EDSR (35)	30.26	0.7621	28.91	0.7121	25.24	0.6904
	SwinIR (36)	33.35	0.8809	32.01	0.8603	31.16	0.8534
	MCSR (5)	32.74	0.8724	31.47	0.8524	28.39	0.8216
	MINet (16)	33.52	0.8843	32.24	0.8643	30.59	0.8607
	McMRSR (8)	33.79	0.8850	32.43	0.8650	30.97	0.8646
	HAT (19)	34.30	0.9023	33.18	0.8904	32.98	0.8712
	**DAASR(Ours)**	**34.95**	**0.9097**	**33.43**	**0.8968**	**33.41**	**0.8919**
Brain(ours)	Bicubic	27.26	0.7920	26.91	0.7771	26.21	0.7504
	SRCNN (34)	30.74	0.8424	29.48	0.8223	28.69	0.8102
	EDSR (35)	31.57	0.8894	30.27	0.8714	29.01	0.8418
	SwinIR (36)	35.26	0.9520	34.37	0.9421	34.06	0.9221
	MCSR (5)	35.84	0.9672	35.01	0.9502	31.89	0.9346
	MINet (16)	36.53	0.9734	35.54	0.9627	34.34	0.9438
	McMRSR (8)	36.86	0.9749	35.82	0.9629	34.76	0.9452
	HAT (19)	36.93	0.9831	35.97	0.9711	35.81	0.9515
	**DAASR(Ours)**	**37.38**	**0.9863**	**36.31**	**0.9763**	**36.64**	**0.9571**

Best results are highlighted in bold. All results are computed under the same evaluation protocol, and reference images are selected within the same data split to avoid cross-split contamination.

### 4.3 Comparisons with state-of-the-art methods

To evaluate the effectiveness of the proposed reference-guided MRI super-resolution framework, we conduct comprehensive quantitative and qualitative comparisons with representative super-resolution methods on both FastMRI and Brain (ours) datasets. The compared methods include classical interpolation-based and CNN-based single-image super-resolution (SISR) approaches, Transformer-based models, as well as recent reference-based and multi-contrast MRI super-resolution methods. All methods are evaluated under identical experimental settings using PSNR and SSIM as quantitative metrics, with scaling factors of ×2, ×3, and ×4. For qualitative evaluation, visual comparisons are conducted at the most challenging ×4 scale to highlight differences in anatomical detail preservation and structural consistency.

#### 4.3.1 Quantitative comparisons

The quantitative results on the FastMRI and Brain (ours) datasets are summarized in Table 1. Overall, the proposed DAASR consistently achieves superior performance across all scaling factors and datasets, demonstrating the effectiveness of reference-guided dual-attention model for MRI super-resolution.

On the FastMRI dataset, DAASR outperforms all competing methods at ×2, ×3, and ×4 scales in both PSNR and SSIM. Compared with classical SISR methods such as SRCNN (34) and EDSR, DAASR shows substantial gains, particularly at higher magnification factors, where conventional methods suffer from pronounced structural degradation. For example, at ×4, DAASR achieves a PSNR of 33.41 dB, significantly exceeding EDSR (35) and SwinIR (36), indicating improved robustness under severe resolution loss. When compared with advanced Transformer-based methods and reference-based MRI super-resolution approaches, including SwinIR, MINet (16), McMRSR (8), and HAT (19), DAASR maintains a consistent advantage. Notably, although McMRSR achieves strong performance by leveraging reference information, DAASR further improves PSNR and SSIM at all scales, suggesting that the proposed hierarchical feature alignment and dual-attention aggregation enable more effective utilization of reference cues while suppressing reference-induced inconsistencies.

Results on Brain (ours). The performance gains of DAASR are more pronounced on the Brain (ours) dataset, which consists of real clinical brain MRI data with complex anatomical structures. Across all scaling factors, DAASR achieves the highest PSNR and SSIM values among all compared methods. At ×4, DAASR reaches 36.64 dB PSNR and 0.9571 SSIM, outperforming the strongest baseline HAT by a clear margin. Compared with SISR methods, the improvement is particularly significant, highlighting the limitation of single-image models in preserving fine brain anatomical details under large upscaling factors. Even compared with recent reference-based and multi-contrast MRI super-resolution methods such as MINet and McMRSR, DAASR consistently delivers better quantitative results. This indicates stronger robustness to variations in reference quality and improved structural consistency, which are critical for reliable clinical brain MRI reconstruction.

Overall, the quantitative results demonstrate that DAASR effectively balances reference-guided detail enhancement and structural fidelity, leading to consistent performance gains across different datasets and scaling factors.

#### 4.3.2 Qualitative comparisons

**Figure 4:**
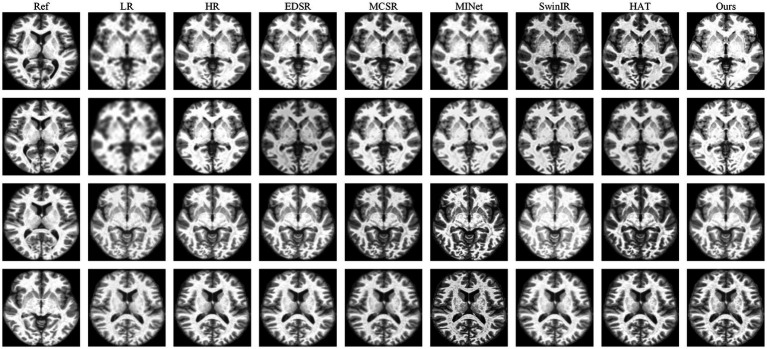
Qualitative comparison on our Brain (ours) dataset under ×4 scales with skull stripping. Compared with representative baselines (EDSR, MCSR, MINet, SwinIR and HAT), our method produces sharper cortical boundaries, clearer sulcal lines, and richer vessel-like textures, while effectively suppressing artifacts.

**Figure 5:**
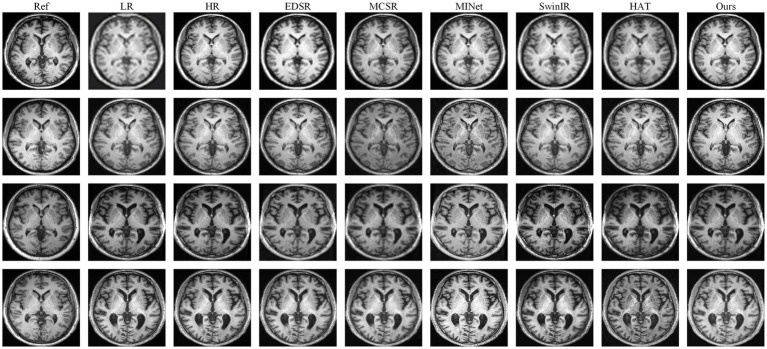
Qualitative comparison on our Brain (ours) dataset under ×4 scales without skull stripping. Compared with representative baselines (EDSR, MCSR, MINet, SwinIR, and HAT), our method produces sharper cortical boundaries, clearer sulcal lines, and richer vessel-like textures, while effectively suppressing artifacts.

Figures 4, 5 show visual comparisons on our Brain (ours) dataset across four representative slices. The first two rows correspond to cases without skull stripping, while the third and fourth rows are from skull-stripped images. Across all settings, our method consistently generates reconstructions that are sharper, more structurally faithful, and closer to the ground truth than those produced by existing approaches.

For the non–skull-stripped slices (first and second rows), most baseline methods struggle to recover the delicate cortical and subcortical structures. EDSR and MCSR often produce oversmoothed reconstructions in which the cortical folds are indistinct and the gray–white matter interface appears blurred. MINet partially enhance structural boundaries but tend to generate blocky artifacts or lose continuity along thin-layer tissues. SwinIR and HAT improve edge sharpness, yet the sulcal contours and thin cortical ribbons remain incomplete, leading to a loss of diagnostic detail. In contrast, our method reconstructs the gyri and sulci with noticeably higher fidelity: the cortical boundaries are continuous, the transitions between white and gray matter are smoother and more anatomically plausible, and fine-grained texture around the cortex is faithfully restored. These improvements highlight the model’s ability to capture subtle spatial variations while maintaining global consistency.

For the skull-stripped slices (third and fourth rows), the difficulty lies in reconstructing subtle internal structures once the surrounding skull context is removed. Here, competing methods show different weaknesses, SwinIR tend to oversmooth vascular and sulcal details, producing homogeneous textures that obscure clinically relevant features; MINet frequently introduces irregular contrast across local regions; and HAT, while sharper, still misses fine vessel-like patterns and occasionally amplifies noise near tissue boundaries. Our approach, however, produces clearer and more continuous sulcal lines, faithfully preserves small vascular-like textures within the white matter, and avoids artificial ringing or halo artifacts around edges. Notably, the contrast between adjacent tissues is better maintained, making the reconstructed slices not only visually sharper but also more anatomically interpretable.

The superiority of DAASR in MRI super-resolution stems from its ability to separate and refine structural and textural details through its dual-attention aggregation mechanism. The Channel-Wise Transformer (CWT) preserves anatomical structure by capturing long-range dependencies, while the Spatial Feature Extractor (SFE) and Spatial Alignment Transformer (SAT) modules ensure precise texture alignment from the reference image, maintaining high-frequency detail. This combination allows DAASR to recover fine cortical structures and tissue boundaries more accurately than existing methods, particularly in challenging scenarios like skull-stripped MRI slices. Its hierarchical refinement approach ensures consistent performance across scales, making it ideal for clinical applications where both structural fidelity and texture clarity are crucial for diagnosis.

### 4.4 Ablation study

Further validation of the effectiveness of our modules and design strategies is provided through a series of ablation experiments conducted in this section. These studies cover the contributions of key components, overall architectural design, loss function configurations, and several important hyperparameter settings. Through systematic comparisons on multiple datasets, we aim to highlight the practical impact of each design choice within the DAASR framework.

#### 4.4.1 Effectiveness of HFAG

To investigate the contribution of the Hierarchical Feature Alignment Group (HFAG) in the proposed DAASR framework, we conduct ablation experiments on the FastMRI dataset by progressively removing key components within HFAG. Quantitative results under ×2, ×3, and ×4 upscaling factors are reported in Table 2.

**Table 2:** Results of the proposed DAASR framework on the FastMRI dataset.

Scale		2×		3×		4×	
Dataset	Variant	PSNR	SSIM	PSNR	SSIM	PSNR	SSIM
FastMRI	w/o CWT&SAT	34.40	0.9031	33.17	0.8894	32.82	0.8803
	w/o CWT	34.63	0.9044	33.26	0.8905	33.09	0.8821
	w/o SAT	34.24	0.8973	32.87	0.8826	32.60	0.8732
	**DAASR(ours)**	**34.95**	**0.9097**	**33.43**	**0.8968**	**33.41**	**0.8919**

PSNR (dB) and SSIM are reported for different upscaling factors (×2, ×3, and ×4) to evaluate the effectiveness of the Hierarchical Feature Alignment Group (HFAG) and its key components.

Best results are highlighted in bold.

HFAG is designed to hierarchically integrate structural modeling and reference-guided detail alignment through repeated aggregation of CWT- and SAT-based modules. By progressively refining feature representations, HFAG enables stable reconstruction of anatomical structures across different spatial scales. As shown in Table 2, removing both CWT and SAT (w/o CWT & SAT) leads to a noticeable performance degradation across all scaling factors, with a particularly evident drop at ×4. This indicates that hierarchical aggregation alone, without explicit structural modeling or reference alignment, is insufficient to preserve fine anatomical details in MRI super-resolution. When only the structural modeling branch is removed (w/o CWT), the reconstruction quality decreases consistently compared with the full DAASR model. The reduction in PSNR and SSIM suggests that CWT plays a critical role in maintaining global structural consistency, which is essential for preserving coherent anatomical layouts in MRI slices. Without CWT, the network tends to lose long-range dependencies, resulting in subtle but cumulative distortions of tissue boundaries under large upscaling factors. Similarly, removing the spatial alignment transformer (w/o SAT) causes a more pronounced degradation, especially at ×3 and ×4 scales. This highlights the importance of accurate reference-guided alignment within HFAG. In the absence of SAT, reference information cannot be reliably aligned with the target anatomy, leading to mismatched structural cues and reduced reconstruction fidelity. Such misalignment is particularly detrimental in MRI, where small structural inconsistencies may affect the interpretability of anatomical regions.

In contrast, the full DAASR model, which incorporates HFAG with both CWT and SAT, achieves the best performance across all scales. The consistent improvements demonstrate that HFAG effectively coordinates structural modeling and reference-guided alignment in a hierarchical manner, enabling robust reconstruction of anatomically faithful MRI images. These results confirm that HFAG is a crucial component for stabilizing reference-based MRI super-resolution, especially in high-magnification scenarios where accurate preservation of anatomical structures is critical for downstream clinical analysis and segmentation-related tasks.

#### 4.4.2 Effectiveness of CWT

To further investigate the role of structural model in DAASR, we evaluate the effectiveness of the Channel-Wise Transformer (CWT) by comparing it with several representative alternatives commonly used for feature aggregation. Specifically, we replace CWT with conventional convolutional layers (CWT-to-Conv), channel attention using SE blocks (CWT-to-CA), and spatial self-attention based on (shifted) window multi-head self-attention (CWT-to-S(W)MSA). Quantitative results on the FastMRI dataset under ×2, ×3, and ×4 upscaling factors are reported in Table 3.

**Table 3:** Results on the FastMRI dataset comparing different structural model strategies.

Scale		2×		3×		4×	
Dataset	Variant	PSNR	SSIM	PSNR	SSIM	PSNR	SSIM
FastMRI	CWT-to-Conv	34.10	0.9005	32.74	0.8823	31.96	0.8699
	CWT-to-CA(SE Block)	34.68	0.9069	33.23	0.8923	32.67	0.8823
	CWT-to-S(W)MSA	34.76	0.9084	33.01	0.8937	32.93	0.8871
	**DAASR(ours)**	**34.95**	**0.9097**	**33.43**	**0.8968**	**33.41**	**0.8919**

PSNR (dB) and SSIM are reported under upscaling factors of ×2, ×3, and ×4 to evaluate the effectiveness of the proposed Channel-Wise Transformer (CWT).

Best results are highlighted in bold.

As shown in Table 3, replacing CWT with standard convolutional layers leads to the most significant performance degradation across all scales. This result indicates that purely local feature model is insufficient for capturing the global structural dependencies required for accurate MRI super-resolution, particularly under large magnification factors. Without explicit long-range structural model, the reconstructed images tend to lose coherence in anatomical layouts, resulting in degraded tissue boundaries and reduced structural continuity. When CWT is replaced with channel attention based on SE blocks, the reconstruction quality improves compared with the convolutional baseline but still remains inferior to the full DAASR model. Although channel attention can reweight feature responses adaptively, it lacks the ability to model inter-channel relationships in a context-aware manner. As a result, structural consistency is only partially preserved, especially in regions with complex anatomical organization. Replacing CWT with spatial self-attention using (shifted) window-based multi-head self-attention further improves performance, demonstrating the benefit of incorporating non-local interactions. However, spatial self-attention primarily focuses on spatial token relationships and may introduce unnecessary sensitivity to local alignment variations. In MRI super-resolution, such spatially driven attention can lead to subtle structural inconsistencies when anatomical patterns vary across slices or subjects.

In contrast, the proposed CWT achieves the best performance across all scaling factors by model long-range dependencies in the channel dimension. This design allows DAASR to capture global structural priors while maintaining stable spatial representations, which is particularly important for preserving anatomically meaningful structures in MRI images. The consistent improvements demonstrate that CWT provides a more suitable structural model strategy than conventional convolutional, channel attention, or spatial self-attention mechanisms for reference-based MRI super-resolution. Overall, these results confirm that CWT plays a critical role in enforcing global structural consistency and contributes significantly to the robustness of DAASR, especially at higher upscaling factors where accurate reconstruction of anatomical structures is most challenging.

#### 4.4.3 Effectiveness of SAT

To assess the effectiveness of the Spatial Alignment Transformer (SAT) for reference-guided MRI super-resolution, we conduct ablation experiments on the FastMRI dataset by modifying the reference alignment strategy. Quantitative results under ×2, ×3, and ×4 upscaling factors are reported in Table 4.

**Table 4:** Results on the FastMRI dataset evaluating different reference alignment strategies.

Scale		2×		3×		4×	
Dataset	Variant	PSNR	SSIM	PSNR	SSIM	PSNR	SSIM
FastMRI	w/o K-V Matching	34.09	0.8955	31.74	0.8793	31.56	0.8629
	w/o F_CWT	34.58	0.8979	32.42	0.8883	32.12	0.8793
	RC to CrossAlign	34.81	0.9013	32.97	0.8913	32.80	0.8863
	**DAASR(ours)**	**34.95**	**0.9097**	**33.43**	**0.8968**	**33.41**	**0.8919**

PSNR (dB) and SSIM are reported under upscaling factors of ×2, ×3, and ×4 to assess the effectiveness of the proposed Spatial Alignment Transformer (SAT).

Best results are highlighted in bold.

As shown in Table 4, removing explicit key–value matching (w/o K–V Matching) leads to a clear performance drop, particularly at higher upscaling factors. This indicates that uncontrolled incorporation of reference information may introduce structurally inconsistent cues, which is undesirable in MRI reconstruction. When the structural modulation component is removed (w/o F_CWT), the reconstruction quality improves but remains inferior to the full model, suggesting that reference alignment without anatomical guidance is insufficient for reliable MRI super-resolution. Replacing SAT with a generic cross-attention–based alignment (RC to CrossAlign) further improves performance but still falls short of the proposed approach. Although generic cross-attention can capture non-local interactions, it lacks explicit awareness of anatomical structure and is therefore less effective in preserving fine tissue boundaries under large magnification. In contrast, the proposed SAT achieves the best performance across all scales by enabling structure-aware reference alignment, allowing complementary information from the reference image to be selectively transferred while suppressing anatomically inconsistent details.

These results demonstrate that SAT is essential for achieving stable and anatomically faithful MRI super-resolution.

#### 4.4.4 Visualization of index and score maps

**Figure 6:**
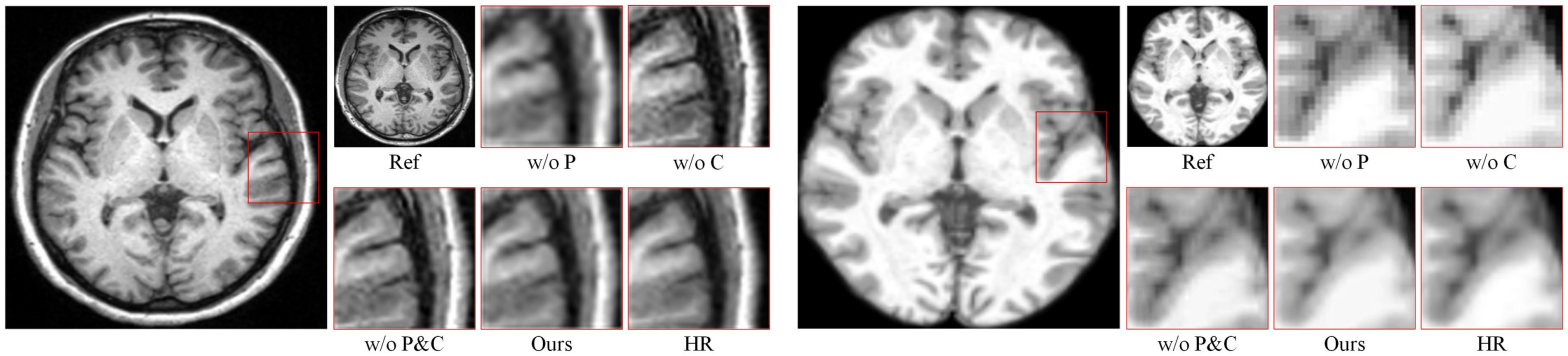
Visualization and ablation study of the index map (P) and score map (C) for reference-guided MRI super-resolution on the Brain (ours) dataset (×4). Two representative cases are shown, including the LR input, reference image, ablation variants, and zoomed-in regions for comparison.

Figure 6 presents qualitative visualizations illustrating the effect of the index map (P) and score map (C) on reference-guided MRI super-resolution. Two representative cases are shown, each including the LR input, reference image, ablation variants, and ×4 zoomed-in details for direct comparison.

When the index map is removed (w/o Index Map (P)), the reconstructed images exhibit noticeable structural discontinuities, particularly in central neural regions. Thin cortical structures are interrupted, forming spurious crack-like artifacts. This suggests that without index-guided correspondence, reference information is aggregated in a less discriminative manner, leading to the suppression of mid-frequency anatomical structures that are essential for maintaining structural continuity in MRI. Removing the score map (w/o Score Map (C)) results in rigid, block-like edges along cortical boundaries. In this case, reference features are uniformly propagated without confidence-aware modulation, causing over-constrained feature responses and reduced smoothness at tissue interfaces. Such artifacts are undesirable in brain MRI reconstruction, as they obscure subtle anatomical transitions and reduce interpretability. When both the index and score maps are removed (w/o P & C), the reconstructions become overly smoothed. Fine sulcal details are blurred, and high-frequency anatomical information is largely lost, indicating that reference guidance degenerates into a weak, non-selective enhancement process.

In contrast, the full DAASR model with both index and score maps (P & C) produces reconstructions with continuous cortical boundaries, well-preserved sulcal patterns, and minimal artificial artifacts. The combined use of index-based correspondence and score-based confidence weighting enables selective and anatomically consistent reference transfer, resulting in textures and structures that are visually closest to the high-resolution ground truth. These results further demonstrate that the proposed design effectively balances detail enhancement and structural fidelity, which is critical for reliable clinical MRI super-resolution.

#### 4.4.5 Effectiveness of patch size

To analyze the influence of patch size on reference-guided MRI super-resolution, we conduct experiments using different patch sizes in the DAASR framework on both FastMRI and Brain (ours) datasets. Quantitative results under ×4 upscaling factors are reported in Table 5.

**Table 5:** Results on the FastMRI and brain (ours) datasets evaluating the effect of different patch sizes.

Dataset	FastMRI		Brain(Ours)	
Patch Size	PSNR	SSIM	PSNR	SSIM
10 × 10	32.76	0.8255	35.97	0.9408
20 × 20	33.03	0.8876	36.22	0.9496
40 × 40	**33.41**	**0.8919**	**36.64**	**0.9571**

PSNR (dB) and SSIM are reported to assess the impact of patch size selection on MRI super-resolution performance.

Best results are highlighted in bold.

Patch size directly affects the granularity at which local structures and reference correspondences are modeled. As shown in Table 5, very small patch sizes tend to limit the receptive field, leading to insufficient modeling of extended anatomical structures and reduced structural consistency, especially at higher magnification factors. Conversely, overly large patch sizes may weaken local detail sensitivity, resulting in suboptimal recovery of fine anatomical features. Across both datasets, a moderate patch size achieves the best overall performance, consistently yielding higher PSNR and SSIM values. This setting provides an effective balance between local detail preservation and global structural coherence, which is critical for MRI super-resolution. Notably, the performance trend remains consistent on the Brain (ours) dataset, indicating that the selected patch size generalizes well to real clinical brain MRI data with complex anatomical patterns.

#### 4.4.6 Effectiveness of loss function

To further analyze the impact of auxiliary loss weighting on MRI super-resolution, we investigate how different combinations of the loss weights *λ*₂ and λ₃ affect reconstruction performance on the FastMRI dataset. Here, λ₁, corresponding to the final reconstruction loss, is fixed to 1, while *λ*₂ (associated with the CWT-based structural loss) and λ₃ (associated with the SAT-based reference alignment loss) are varied. Quantitative results under ×2, ×3, and ×4 upscaling factors are reported in Table 6.

**Table 6:** Results on the FastMRI dataset evaluating the impact of different loss weight settings.

Scale		2×			3×			4×		
$\lambda_{2}$	$\lambda_{3}$	PSNR	SSIM	LPIPS	PSNR	SSIM	LPIPS	PSNR	SSIM	LPIPS
0.1	0.02	34.09	0.8655	0.070	31.74	0.8573	0.084	31.56	0.8529	0.096
0.5	0.01	34.28	0.8690	0.073	31.92	0.8583	0.090	32.12	0.8793	0.102
0.01	0.1	34.56	0.8726	0.077	32.84	0.8653	0.091	32.61	0.8897	0.106
0.3	0.09	34.41	0.8713	0.084	32.67	0.8627	0.097	32.40	0.8863	0.108
0.1	0.01	**34.95**	**0.9097**	**0.065**	**33.43**	**0.8968**	**0.078**	**33.41**	**0.8919**	**0.091**

PSNR (dB), SSIM, and LPIPS (↓) are reported under upscaling factors of ×2, ×3, and ×4 to assess the influence of λ₂ and λ₃ on MRI super-resolution performance.

Best results are highlighted in bold.

In addition to PSNR and SSIM, we report LPIPS as an auxiliary perceptual similarity metric (lower is better) to reflect subtle appearance differences across different loss weight settings. It should be emphasized that LPIPS is used here only for ablation analysis and does not serve as a clinical endpoint. The primary evaluation of MRI reconstruction quality in this work remains focused on structural fidelity and anatomical consistency, as measured by PSNR and SSIM.

The results indicate that the balance between structural supervision and reference-guided supervision plays a critical role in achieving anatomically faithful reconstructions. When *λ*₂ is set to a relatively small value, the model exhibits degraded structural consistency, as reflected by reduced PSNR and SSIM values, particularly at higher upscaling factors. From a medical imaging perspective, insufficient structural regularization may lead to subtle distortions in tissue boundaries and reduced continuity of anatomical regions, which can negatively affect the interpretability of reconstructed MRI images.

Conversely, excessively increasing *λ*₃ places disproportionate emphasis on reference-guided detail transfer. While this setting may slightly reduce LPIPS, it is often accompanied by decreased PSNR and SSIM, indicating the introduction of structurally inconsistent patterns when the reference image is not perfectly matched to the target anatomy. Such behavior is undesirable in MRI super-resolution, as it may produce anatomically implausible details that could interfere with reliable image interpretation or downstream analysis. A moderate combination of λ₂ and λ₃ consistently yields the best overall performance across all metrics. This setting achieves an effective balance between enforcing global anatomical structure through CWT-based supervision and selectively incorporating reference information via SAT. As a result, the reconstructed images preserve continuous tissue boundaries and anatomically coherent layouts while avoiding over-smoothed or artificially enhanced patterns. The concurrent improvement in PSNR, SSIM, and LPIPS further suggests that balanced supervision benefits both structural accuracy and visual consistency without compromising anatomical plausibility.

Overall, this analysis demonstrates that appropriate weighting of structural and reference-guided loss terms is essential for achieving reliable and anatomically faithful MRI super-resolution. Such balanced supervision not only improves quantitative reconstruction accuracy but also enhances the stability and interpretability of the reconstructed images, which is particularly important for downstream tasks such as tissue segmentation and morphological assessment.

## 5 Conclusion

In this work, we proposed a reference-guided MRI super-resolution framework, termed Dual Attention Aggregation Super-Resolution (DAASR), to address the limitations of single-image MRI super-resolution under large upscaling factors. By explicitly balancing structural modeling and controlled reference integration within a hierarchical refinement framework, DAASR enables stable reconstruction of anatomically meaningful details while suppressing unreliable reference-induced artifacts. Extensive experiments on both a public MRI benchmark and a clinical brain MRI dataset demonstrate that the proposed method consistently outperforms state-of-the-art approaches in terms of quantitative accuracy and structural fidelity, while better preserving fine anatomical structures and tissue boundaries. Ablation studies further validate the effectiveness of each key component in enhancing reconstruction stability and anatomical consistency. Future work will explore extending the proposed framework to three-dimensional MRI volumes and integrating it with downstream clinical tasks such as segmentation and quantitative analysis.

## Data Availability

The data analyzed in this study is subject to the following licenses/restrictions: the study utilizes two datasets. (1) The fastMRI dataset is a publicly available resource (available at https://fastmri.med.nyu.edu/). (2) The private clinical dataset was obtained from Gansu Provincial Hospital and is not publicly available due to institutional restrictions and the need to protect patient privacy and confidentiality. Data may be made available from the corresponding author upon reasonable request and with permission from the institutional ethics committee. Requests to access these datasets should be directed to wanglj@ldxy.edu.cn.
